# Green recovery of vanadium from spent vanadium catalyst from sulfuric acid production

**DOI:** 10.1038/s41598-026-47112-6

**Published:** 2026-04-20

**Authors:** Ashraf A. Shaltout, Rabeia S. F. El-Hallag, Taha M. A. Razek

**Affiliations:** 1 Post-graduate student, Faculty of graduate studies and environmental research, Ain Shams university, Cairo, Egypt; 2 Chemistry Department, Faculty of Science, Al-Azhar University, Cairo, Egypt; 3 Environmental Basic Sciences Department, Faculty of Graduate Studies and Environmental Research, Ain Shams University, Cairo, Egypt

**Keywords:** Vanadium, Recovery, Citric acid, Spent catalyst, Sulfuric acid, Chemistry, Environmental sciences, Materials science

## Abstract

Research on extracting secondary raw materials from hazardous waste is gaining global importance. Spent catalysts containing heavy metals pose a great threat to environment. A key example is spent catalysts from sulfuric acid production, which contain 4.66% V_2_O_5_. This work aims at recovering vanadium from spent catalyst discharged from sulfuric acid industry. Experiments used four different leaching acids: tartaric acid, oxalic acid, acetic acid, and citric acid. The effects of temperature, reagent concentration, leaching duration, and solid/liquid (S/L) ratio were tested. Under optimal conditions, an impressive 95% of vanadium was recovered through citric acid leaching, with the following parameters (2% S/L ratio, 1 M citric acid, 70 °C, pH, and a leaching duration of 120 min). Results indicated the potential for recovering vanadium pentoxide from spent vanadium catalysts used in the sulfuric acid industry in Egypt.

## Introduction

The production of sulfuric acid is an important process because of its many applications. Sulfur dioxide (SO_2_) is oxidized to SO_3_ in preparation for sulfuric acid (H_2_SO_4_) making. Oxidation is done by blowing clean dry SO_2_ gas down through vanadium catalyst^1^. Sulfuric acid is one of the most important chemicals worldwide. Sulfuric acid has many applications, in the manufacture of dyestuff, explosives and lead-acid batteries for vehicles. Additionally, it is used in the petroleum refining industry and its derivatives, and in the synthesis of mineral acids such as hydrochloric acid. The major application is the production of fertilizers, including ammonium superphosphate (acid phosphate) and sulphate^2^.

Catalysis is characterized as a mechanism through which the rates of chemical reactions are modified by the introduction of a catalyst. When the catalyst’s activity falls below an acceptable threshold, it is typically regenerated and reused; however, regeneration is not always feasible^3,4^***.*** After several cycles of regeneration and reuse, the activity of the catalyst may diminish, making further regeneration economically unviable^5^. The U.S. Environmental Protection Agency categorizes spent catalysts as hazardous waste^6^.

Vanadium is found in nature primarily as a secondary component within V-containing minerals; however, there are very few natural minerals where vanadium is the dominant element^7^. The US Geological Survey reports that the global vanadium reservoir is estimated to be around 63 million tons, with significant concentrations located in South Africa, China, Australia, and Russia^8^. Consequently, the total global production of vanadium reached approximately 102,365 tons in 2019, with an estimated 75–85% sourced from V-Ti magnetite^9^. It is estimated that roughly 65 × 10^3^ tons of vanadium enter the ecosphere each year from natural processes such as crustal weathering and volcanic emissions, while about 2.3 × 10^8^ kg are released due to various human activities^8,10^.

The production of sulfuric acid (H_2_SO_4_) generates approximately 40 thousand tons of spent vanadium catalyst (SVC) each year on a global scale^11^. In 2022, the global market volume for sulfuric acid reached 265.05 million metric tons. The extraction of vanadium pentoxide (V_2_O_5_) from used catalysts is critically significant from industrial, economic, and environmental perspectives^11^. Vanadium pentoxide (V_2_O_5_) is a substance of considerable importance, possessing vital technological applications owing to its remarkable electronic, magnetic, and catalytic characteristics^12^. Nevertheless, substantial quantities of discarded catalysts are released into the environment as industrial waste^8^. Furthermore, the buildup of discarded vanadium catalysts poses a significant environmental issue^13^. The atmosphere above the accumulated waste contains vanadium pentoxide dust, which is dispersed to nearby areas by the wind. Vanadium pentoxide is known to have detrimental effects on humans, animals, and plants^14^.

The recycling of hazardous materials into high-value products is increasingly recognized in modern society, as it significantly reduces environmental damage while creating valuable resources. To the best of our knowledge, this is the first research conducted on leaching process of vanadium from a solid spent catalyst of sulfuric acid production in Egypt. The main objective of this research was to investigate the extraction of vanadium from the discarded vanadium catalyst. Furthermore, another objective of this study was to discover a leaching reagent that is environmentally benign or nearly so, with minimal negative environmental impacts.

## Experimental

### Materials

Spent catalyst mass used in the manufacture of sulfuric acid was supplied by Egyptian Financial and Industrial Company, Kafr Elzayat, Egypt. The catalyst was provided by the company after the turnover finished, and it was discharged as waste. The spent vanadium catalyst samples were composed of 6 mm average diameter with 20 mm length hexagonal prism structures. The samples were ground by using a planetary ball mill and sieved to isolate particles of ≤ 50 µm diameter. Samples (5–25 g) were used in leaching experiments, keeping the used volume of leaching acid at 25.0 mL. All organic acids, ammonium sulfate, sodium hydroxide were analytical grade Merck products.

### Apparatus

All leaching tests were carried out in a beaker with a magnetic stirrer (Model F60 FALC Instruments, Italy) placed in a thermostat to control the temperature. The mixtures were filtered under a vacuum unit. XRD pattern has been analyzed by X-Pert Pro X-ray diffractometer (CuKα, λ = 1.5418 Å) with the range of 10–80°. pH measurements were carried using an Orion digital pH/mV meter (model SA 720). The determination of vanadium was carried out by FAAS using a GBC Scientific Equipment model (Savant AA). Fourier transform infrared (FT-IR, Bruker ALPHA II, Germany) was used to acquire the infrared spectra. The elemental composition was checked by using an Apollo X SDD energy-dispersive spectrometer (EDX) and Rigaku Supermini200 sequential wavelength dispersive X-ray fluorescence (WDXRF) Spectrometer.

### Leaching methods

The leaching processprocess employed an organic acidic medium to extract vanadium from the spent catalyst. Citric acid, oxalic acid, acetic acid, and tartaric acid were utilized for the leaching process of vanadium. Each leaching experiment was conducted simultaneously under identical conditions by the same individual, utilizing 250 mL and/or 500 mL clean and dry Erlenmeyer flasks. These flasks were filled with various measured masses of spent vanadium catalyst (SVC) along with a specific volume of the leaching acid, which was measured using a graduated cylinder and subsequently added to the flasks; these flasks were then stirred using a multi-stage hot plate equipped with a magnetic stirrer. To examine this, the concentration of citric acid was varied between 0.1 and 1.0 M in this study, while the other parameters (S/L: 2%, leaching time: 120 min, temperature: 25°C) remained constant.

To study the effect of the contact time, the vanadium leaching was performed at different times, from 60 to 180 min, under the following conditions: [citric acid]-1M, S/L: 2 (w/v)%, and temperature 25°C.

Leaching experiments were carried out at different solid-to-liquid (S/L) ratio (g of spent catalyst/mL of leaching acid) ranging from 2 to 4% to investigate the effect of S/L ratio while the other parameters were fixed.

The effect of pH in the range of 1.7–5 on vanadium leaching was studied while keeping other experimental parameters such as citric acid concentration, temperature, time, and S/L ratio at 1M; 25°C, 120 min and 2%.

**Fig. 1:**
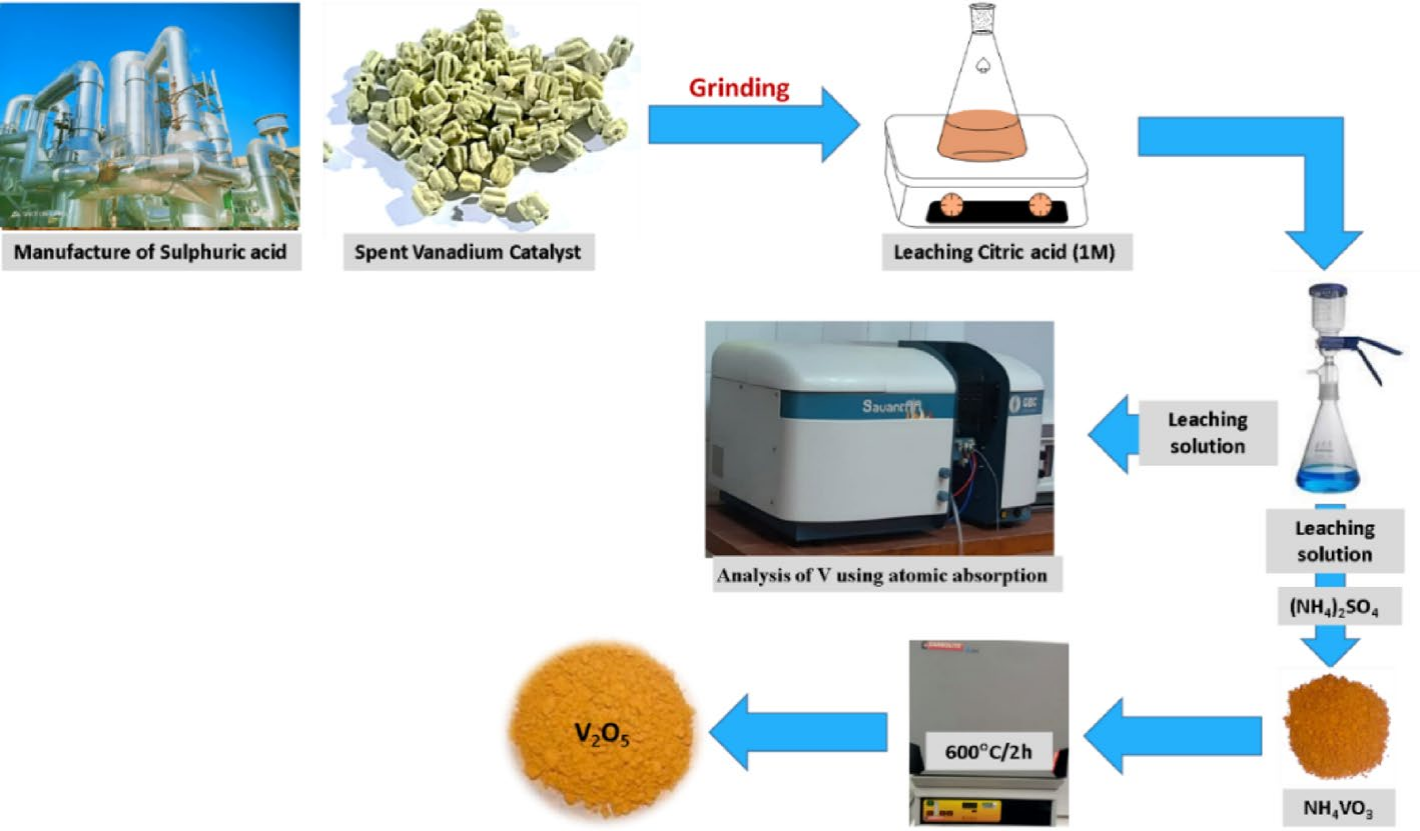
Schematic representation of the recovering of vanadium from SVC.

To investigate the effect of temperature, leaching experiments were carried out at 25°C, 50°C and 70°C while the other parameters remain constant. Following this, the flasks underwent vacuum filtration to collect both the solid residue and the resulting leaching solution. A schematic illustration of the treatment steps of SVC and the subsequent analysis is presented in Fig. 1.

By calculating the concentration of vanadium in the solution and using Eq. (1) to relate the recovered vanadium to the nominal value contained in the solid, the leaching efficiency was assessed.

1 $$\%Recovery \,of \,vanadium = \frac{Vanadium \,concentration \,in \,the \,solution \times V}{mass \,of \,V-ion \,in \,the \,spent \,catalyst} \times 100$$

where V is the volume of leaching solution (in mL). The concentration of vanadium in the solution was determined using atomic absorption spectrophotometry.

## Results and discussion

### Characterization of the spent vanadium catalyst (SVC)

**Fig. 2:**
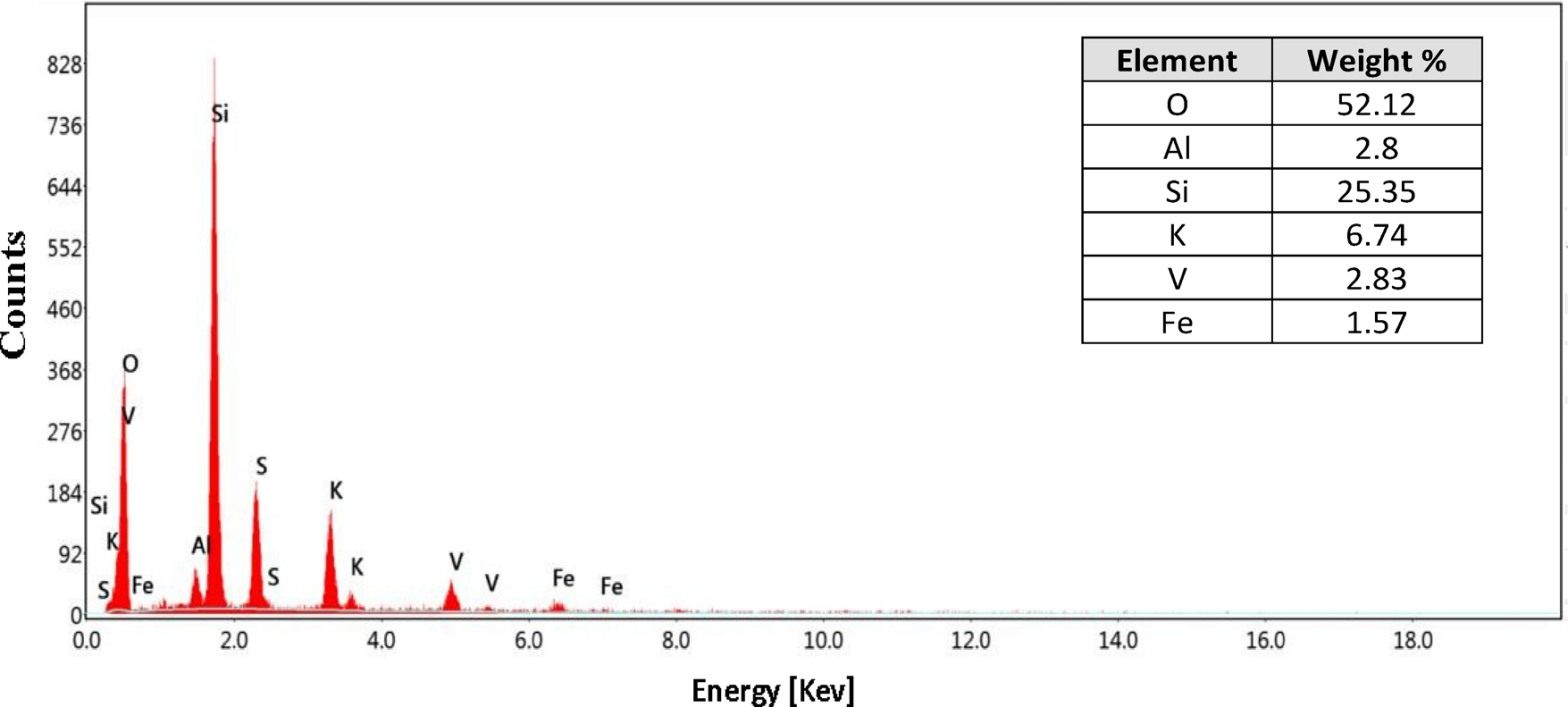
EDX spectra for spent vanadium catalyst (SVC).

The elemental composition of the SVC is reported in Table 1 and Fig. 2. Vanadium penta oxide concentration is 4.66% w/w, while SiO_2_ and Al_2_O_3_ content is 30.9% and 4.6%, respectively.

**Table 1:** Main chemical analysis of SVC.

Constituents	Al_2_O_3_	SiO_2_	P_2_O_5_	SO_3_	K_2_O	V_2_O_5_	Fe_2_O_3_	Na_2_O	CaO	TiO_2_
Composition (w/w) %	1.95	45.10	0.22	20.30	8.00	4.66	0.93	1.57	1.19	0.11

**Fig. 3:**
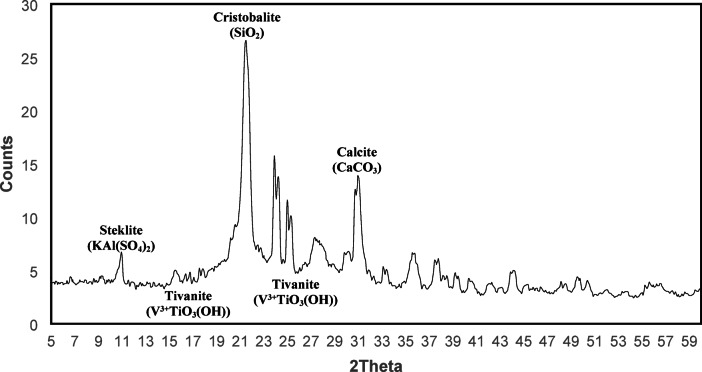
XRD powder diffractogram of spent vanadium catalyst (SVC).

Figure 3 shows the diffractogram for the spent vanadium catalyst (SVC) (after the industrial process). The major peaks found at 2θ 11° for steklite (KAl(SO_4_)_2_), 16° and 25° for tivanite (V^3+^TiO_3_(OH)) reveal the presence of vanadium, 21.5° for cristobalite (SiO_2_), and 31° for calcite (CaCO_3_).

### Key factors affecting leaching process

#### Screening of organic acids on leaching of V from spent catalyst

**Fig. 4:**
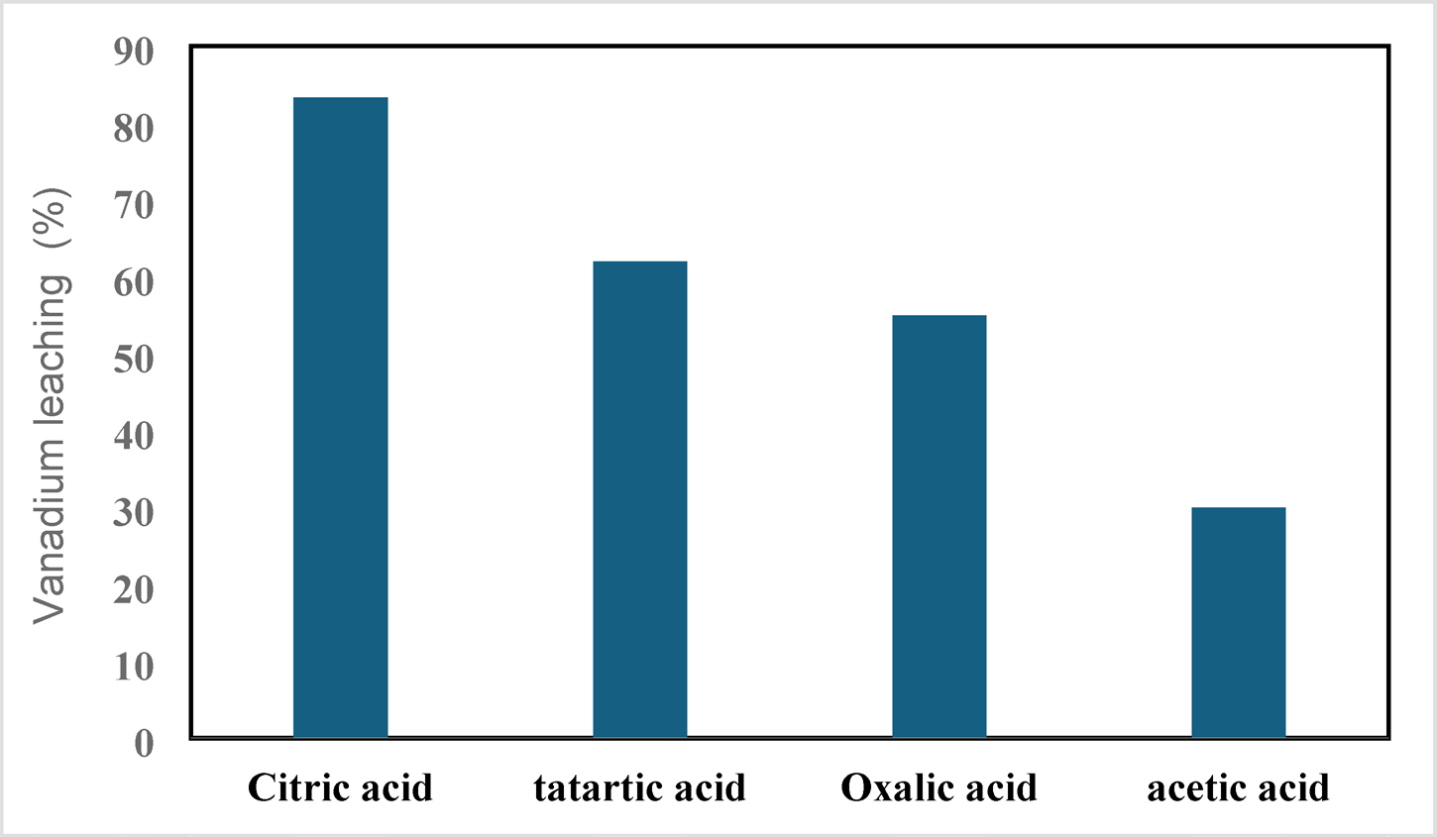
Leaching results of SVC with organic acids.

Due to several benefits, such as low waste release and lower emissions, spent catalyst leaching has generally been investigated using natural organic acids. Four distinct acids were used in a batch acid extraction experiment (25.00 mL of the acid, 0.50 g of waste catalysts, 25.0 °C). The chosen concentration of each of these acids—citric, oxalic, tartaric, and acetic acid—was 1 M. The leaching trend with either of the organic acids is as follows, and the leaching efficiency of V was highest with citric acid, as illustrated in Fig. 4, 83% citric acid, 62% tartaric acid, 55% oxalic acid, and 30% acetic acid are in order. Based on the preceding pattern, citric acid was regarded as a suitable lixiviant in this examination and so examined throughout the leaching study. Results are supported by ***Erust, et al.***^15^.

The findings further indicate that citric acid emerged as the most effective leachant among the acids evaluated, as it resulted in a higher percentage of recovery in comparison to the other acids. Additionally, it is an inexpensive, readily available, and less aggressive acid. This could explain the significant interaction observed between the vanadium ion and the citrate ion. Consequently, a comprehensive leaching study was meticulously conducted by adjusting various operational parameters, including leaching duration, citric acid concentration, solid/liquid ratio (S/L)%, temperature, and pH, specifically for vanadium extraction. The subsequent section provides an in-depth analysis of the leaching outcomes related to the operational factors examined for the extraction of vanadium from the spent catalyst phase.

#### Effect of citric acid concentration on leaching efficiency

**Fig. 5:**
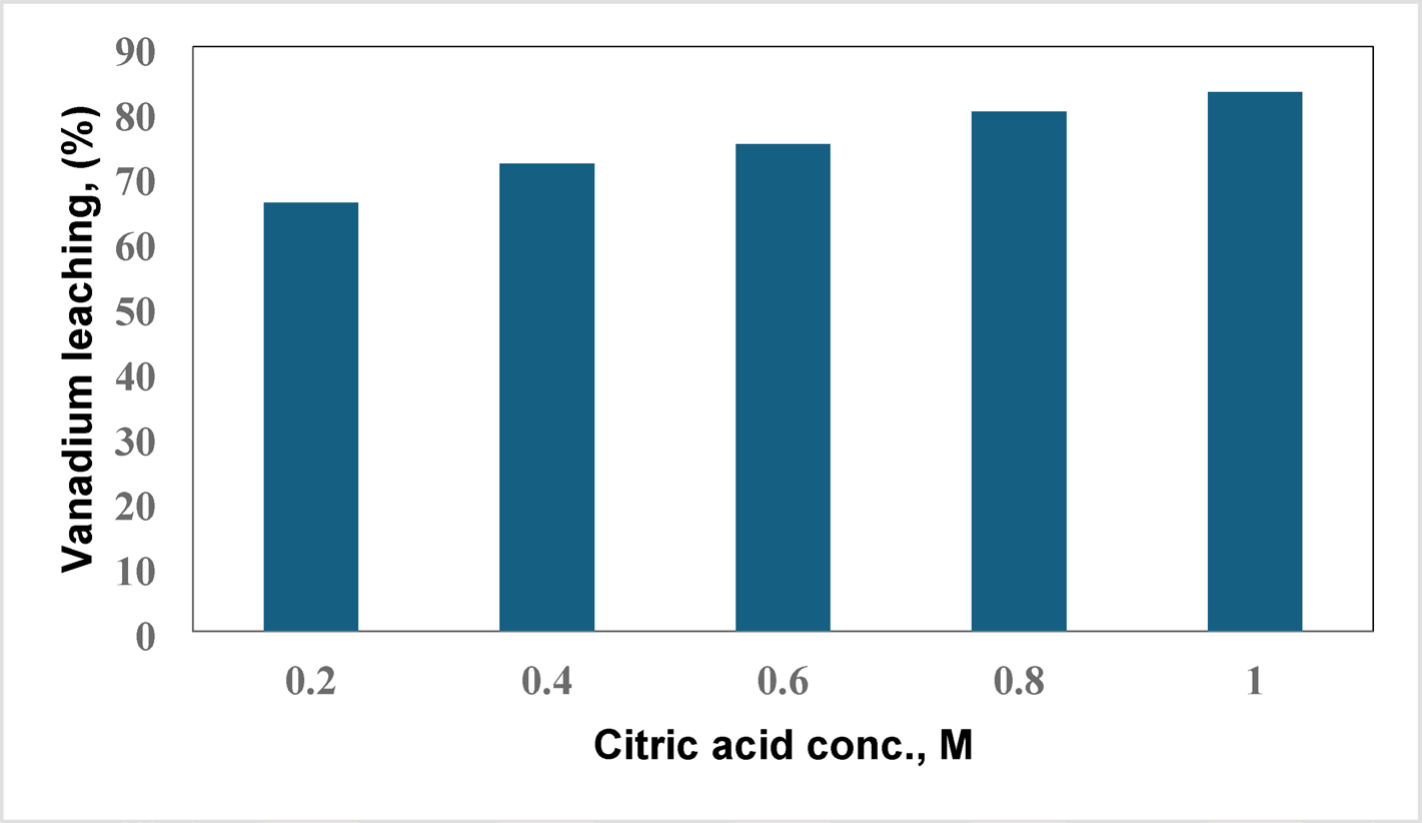
Effect of citric acid concentrations on vanadium recovery.

By comparing the leaching efficiency across various citric acid concentrations, a further examination into the impact of altering concentration of leaching acid on the leaching efficiency was conducted. Using 0.5 g of spent catalyst, a series of citric acid concentrations, namely 0.2–1.0 M, were carefully prepared and then used to leach vanadium from the spent vanadium catalyst. The amount of leached vanadium in response to the concentration of citric acid is depicted in Fig. 5 quite. One of the most important factors in the leaching process for successful metal extraction is the acid concentration. To examine this, the concentration of citric acid was varied between 0.2 and 1.0 M in this study, while the other parameters (S/L: 2%, leaching time: 120 min, temperature: 25°C) remained constant. As seen in Fig. 5, the extraction of V increased from 66 to 83%, causing the acid concentration to change from 0.2 M to 1.0 M. After that, the leaching efficiency remained same. According to this investigation, 1.0 M is sufficient to dissolve vanadium from SVC^15^.

#### Effect of leaching time

**Fig. 6:**
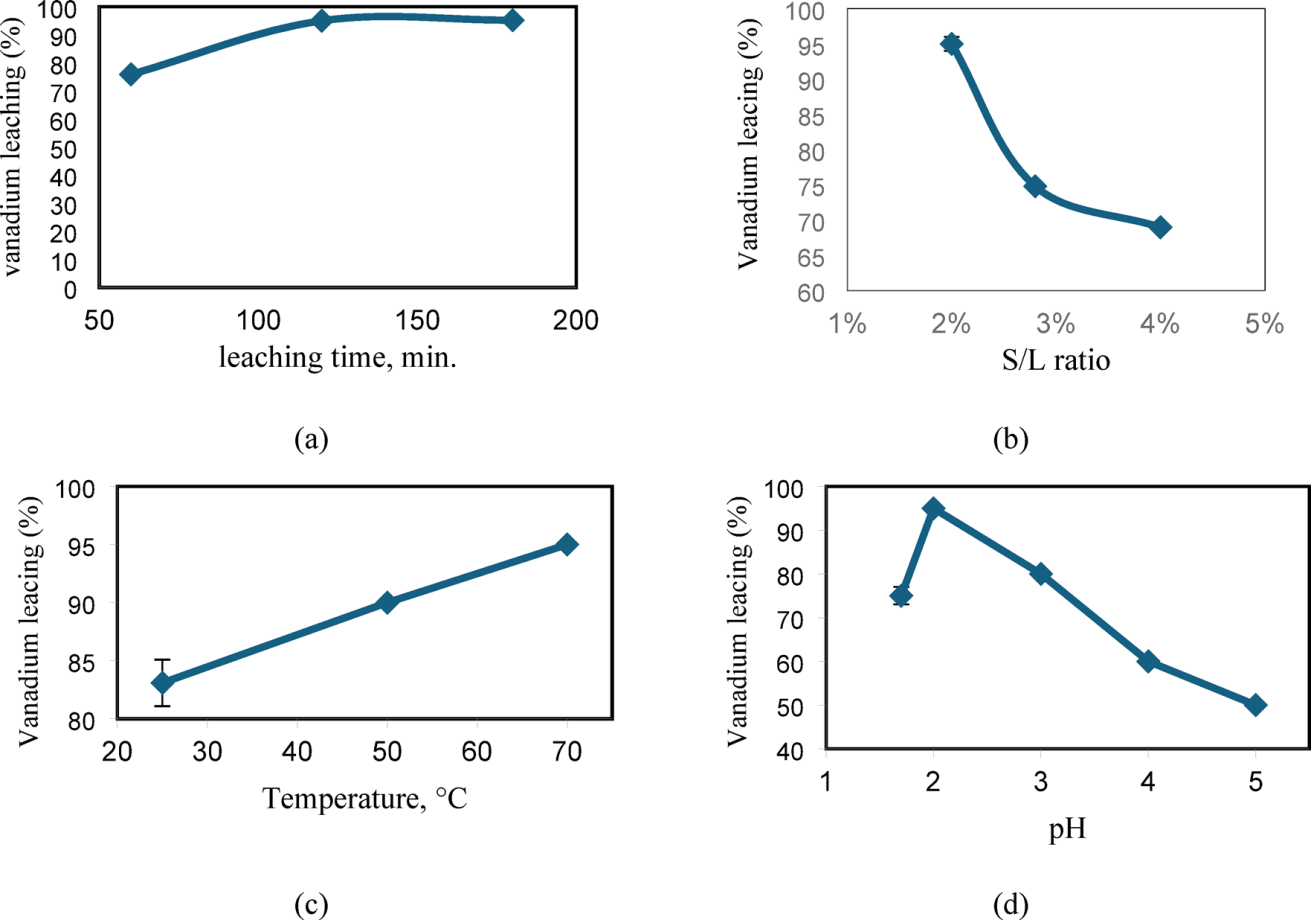
Testing leaching parameters (**a**) Effect of leaching time (**b**) Effect of S/L ratio (**c**) Effect of temperature (**d**) Effect of leaching solution pH on vanadium recovery.

Vanadium leaching from the spent catalyst phase as a function of leaching duration (1–180 min) was studied; the extraction behavior of vanadium over time is depicted in Fig. 6a. The settings for this experimental work were [citric acid]-1M, S/L: 2 (w/v)%, and temperature 25°C. All other parameters were held constant. The preceding figure made it clear that the vanadium leached more quickly, yielding around 70% of its total value after 60 min. As the leaching duration increased to 120 min, the efficiency remained consistent, hence setting the mixing duration at two hours is sufficient. A similar type of leaching with time by was also observed by^9,16^. Therefore, 120 min leaching time seemed to be sufficient to extract appreciable amount of vanadium and was adopted in further studies.

#### Effect of S/L ratio

The recovery of vanadium from spent catalysts depends critically on the S/L ratio. Maximizing recovery efficiency, cutting expenses, and limiting the leaching process’s negative environmental effects all depend on optimizing this ratio. The impact of the S/L ratio on the percentage of vanadium recovered using 1.0 M citric acid is depicted in Fig. 6b. The findings show that at the lowest S/L ratio, the most recovery was achieved. These findings generally concur with comparable systems’ leaching efficiency^17,18^. Increasing the S/L ratio will, in general, decrease diffusion due to the increased viscosity. This will result in a hindrance in the movement, leading to a decreased leaching efficiency. This also could be explained using Eq. (1), shown above: There is an increase in the mass of V_2_O_5_ in the spent catalysts as the mass of the spent catalyst increases; however, on the other hand, the mass of V_2_O_5_ in the spent catalyst is in the denominator of Eq. (1). It is evident that the volume of the recovered sample following leaching (Vf) in the nominator is multiplied by the ppm of V recovered from the tested sample. As a result, the recovery percentage falls as the S/L ratio rises because the denominator rises as the mass of the spent catalyst increases^17,18^.

#### Effect of temperature

By calculating the recovery percentage as a function of extraction temperature between 25°C and 70°C under the same experimental settings, the impact of temperature on vanadium recovery from a spent vanadium catalyst was examined. Figure 6c demonstrates that, with the exception of 50°C, the temperature in the studied range has a minimal effect on the recovery percentage. The temperature selected for the studies in Poland and Turkey is the same as this mentioned by Erust et al.^15^* and Mazurek*^19^***.*** In addition to the impact of mass transfer with diffusion parameters, we believe that the coagulation of the solid spent catalyst may be responsible for this rise in recovery at 70.0°C. Two opposing factors could account for this: the system’s increased dynamics, which tend to raise the recovery percentage as the temperature rises, and the solution’s propensity to coagulate and aggregate, which prevents vanadium from leaching from the spent catalyst^20^.

The reaction temperature affects how metals are leached using citric acid. As the leaching process is an endothermic reaction^21–23^, raising the temperature enhanced the leaching of the metals under study, according to the data shown in Fig. 8. Eighty-three percent of the vanadium leached at room temperature. By raising the reaction temperature from 25 to 70°C, respectively, the leaching efficiencies of V metals rose to 95%. The reaction mechanism is surface area-controlled at room temperature, but as the temperature rises, citric acid diffuses into the grain core and splits into pieces, speeding up the rate at which metals leach^24^***.***

#### Effect of pH

The effect of pH in the range of 1.7–5.0 on vanadium leaching was studied while keeping other experimental parameters are constant. As shown in Fig. 6d, V leaching efficiency increased from 75.0% to 95.0% with the increase in pH from 1.7 to 2.0 and then decreased gradually to 50.0% at pH 5. Vanadium generates various anionic compounds at low pH, hence its extraction efficiency is greatly reliant on pH^25^. The extraction of vanadium exhibits the opposite pattern, with the highest recovery occurring at pH 2.

### Characterization of typical leach residue sample

**Fig. 7:**
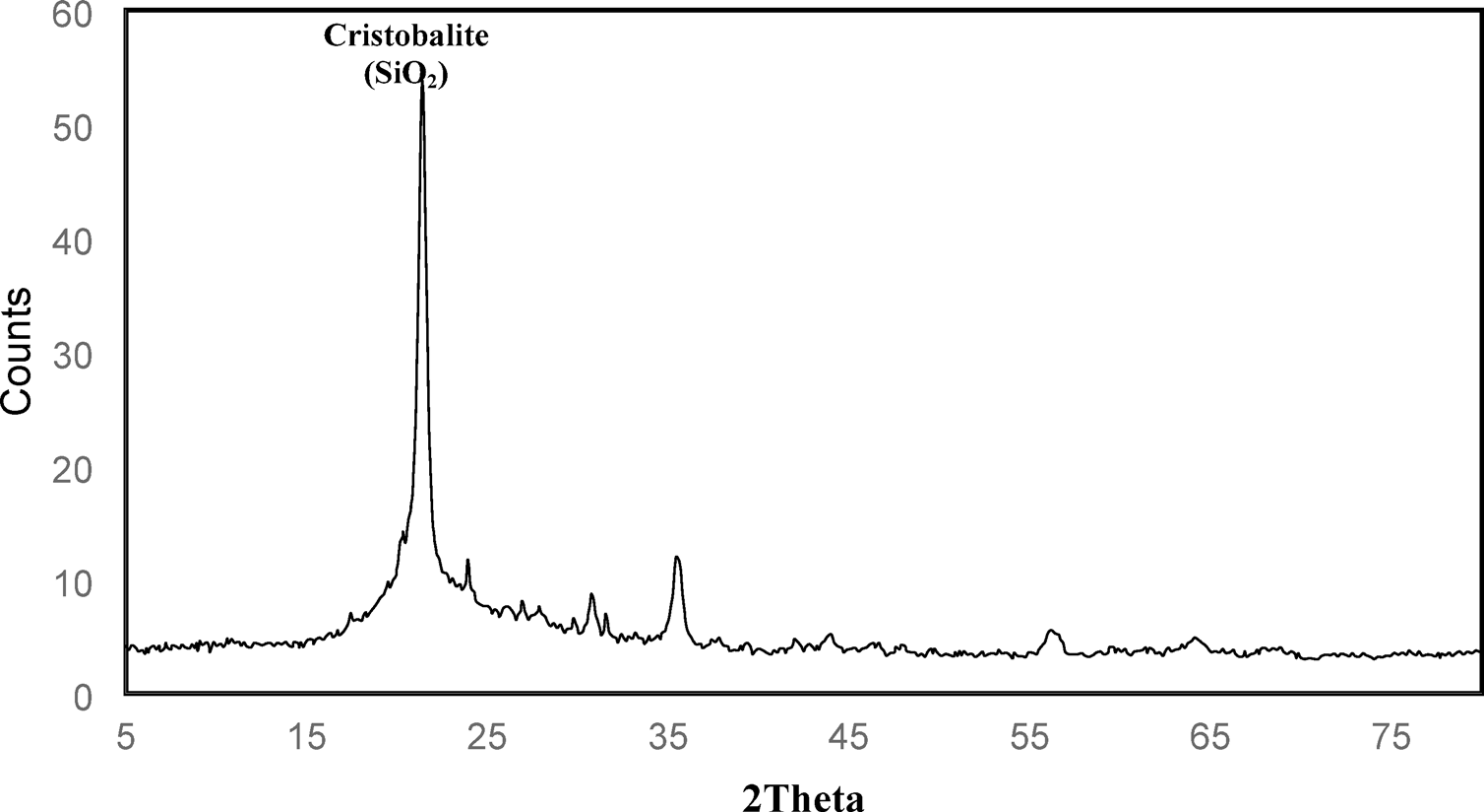
The XRD powder diffractogram of residual solid after leaching of spent vanadium catalyst (SVC).

As shown in Fig. 7, and as expected, the residue recovered following citric acid leaching does not display any peak on the XRD pattern due to vanadium (V) in any form when compared to the original sample. Similarly, the typical peak for V_2_O_5_ was not identified, as evidenced by the XRD analysis results; however, the primary peaks related to SiO_2_ were reported in the XRD data. Thus, full extraction of vanadium from the spent catalyst phase at the optimal condition employing 1.0 M citric acid was clearly demonstrated.

### Leaching mechanism

**Fig. 8:**
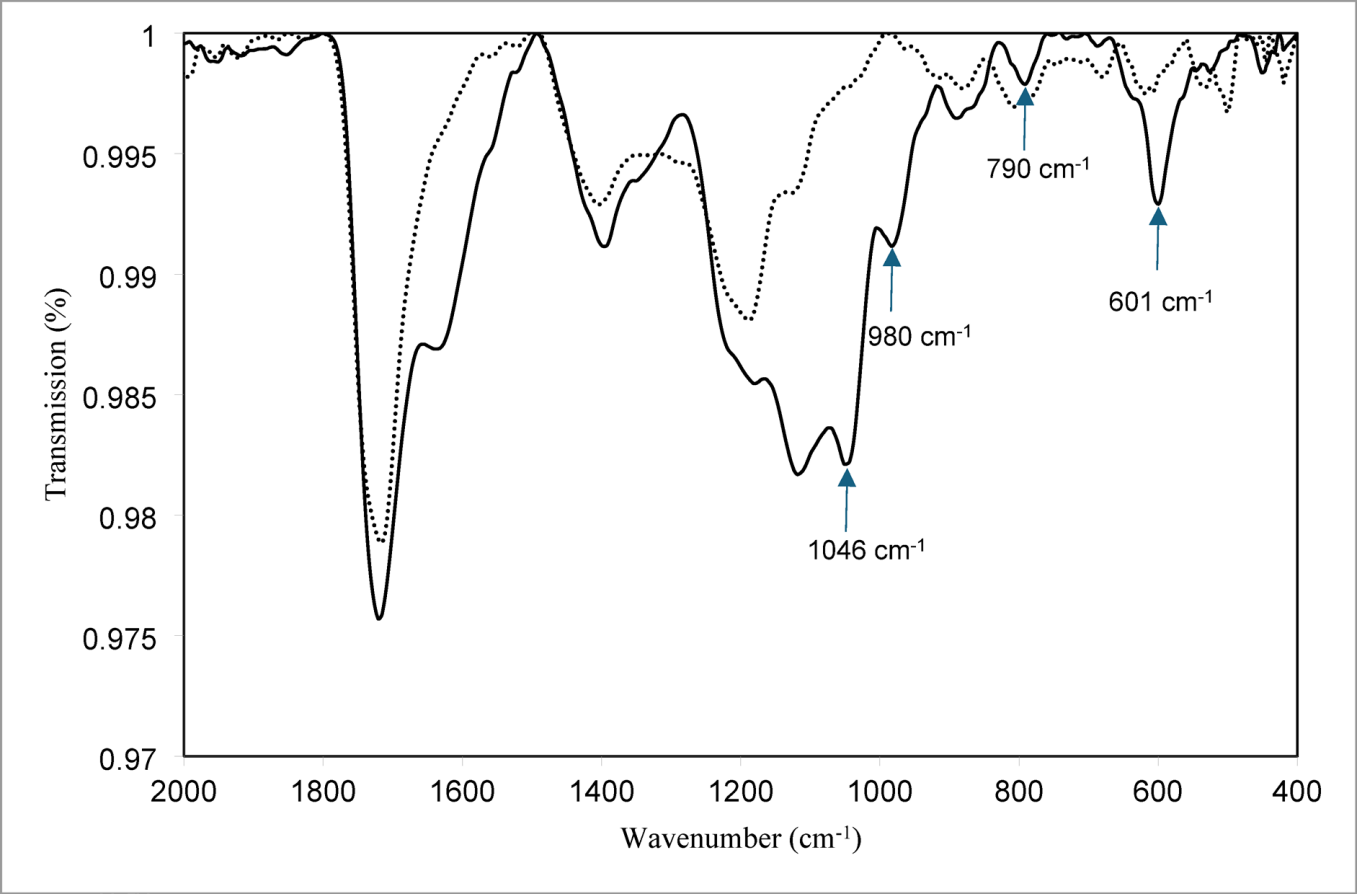
FTIR of citric before (…….) and after (–––) leaching of vanadium from SVC.

To evaluate the changes in citric acid before and after vanadium extraction, Fourier transform infrared (FT-IR) spectra of neat and vanadium-loaded citric acid were recorded. The results are displayed on Fig. 8. At 980 cm^−1^, 790 cm^−1^, and 601 cm^−1^, new peaks emerged following the extraction of vanadium into citric acid. The polyoxovanadate’s V=O stretching vibrations are the cause of the peak at 980 cm^−1^. Furthermore, the symmetric and asymmetric V–O–V stretching seen in decavanadates are shown by bands at 790 cm^−1^ and 601 cm^−1^^26^^,^^27^.

**Fig. 9:**
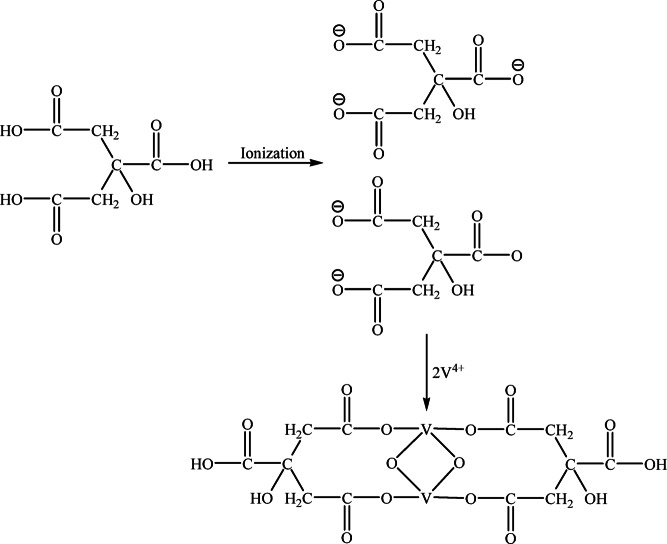
Schematic reaction of citric acid with vanadium.

Attenuated Total Reflectance-Fourier Transform infrared spectroscopy (ATR-FTIR), which gives details about the precise functional groups in a material, was also used to validate the development of the vanadium-citrate complex. The spectrum of real leach liquor shows that the peak at 1046 cm^–1^ was not present in the citric acid spectra. This peak was primarily V=O (vanadyl), which was attributed to the leach liquor’s vanadium’s V^4+^ oxidation state. The presence of the vanadium-citrate complex in the leach fluid was indicated, and the blue coloration validated the V^4+^ condition^28^. Despite being a weak acid, citric acid works well as a metal binder and leaching agent. It is more efficient than mineral acids because it leaches metals through two different processes: the direct displacement of metal ions by hydrogen ions (acidolysis) and the creation of soluble metal complexes. Citric acid and other weak organic acids increase metal solubility by lowering the positive charge of metal cations^29^. Vanadium leaching with citrate involves complexation, where vanadium species interact with citrate in a 2:2 ratio^30^, as shown in the accompanying Fig. 9.

The leaching efficiency of various agents for leaching of vanadium from various spent catalysts is revealed by a literature review (Table 2). Table 2 compares the leaching efficiency of the citric acid to those of similar various leaching agents, highlighting the feasibility of employing citric acid for the leaching of vanadium from SVC.

**Table 2:** Comparison of leaching efficiency and recovered products by various leaching agents for vanadium ion from various spent catalysts.

Leaching agents	Temperature, °C	Leaching efficiency (%)	Spent catalyst	References
H_2_SO_4_	70	98	Linz Donawitz (LD) converter Slag	^31^
H_2_SO_4_	90	95.9	Stone coal	^32^
H_2_SO_4_	80	94.3	Fly ash	^33^
H_2_SO_4_	80	42	Spent catalyst (Used in denitration (Denox) processes for flue gases in power plants)	^34^
HCl/H_2_SO_4_/HNO_3_	-	99	Spent catalyst (in petrochemicals industries)	^35^
NaOH	240	80	Steel slag	^9^
NaOH	-	68.4	Steel Slag	^36^
Acetic acid	-	90.3	V-based Ca Residue	^37^
Oxalic acid	50	~ 90	Spent catalyst	^19^
Citric acid	70	95	Spent catalyst	This work

### Vanadium recovery from leaching liquid

The recovery of vanadium presents a significant challenge, as it not only facilitates the reclamation of this metal, thereby reducing the necessity for imports, but also mitigates the potential environmental harm caused by highly toxic vanadium compounds found in wastewater. To precipitate vanadium as ammonium vanadate, ammonium sulfate (or chloride) was introduced into the leaching solution, in accordance with Eqs. (2, 3). The pH of the resulting solution was approximately 11.5, achieved by the addition of sodium hydroxide, followed by the incorporation of forty grams of ammonium sulfate into 300 mL of leaching liquid, which was then stirred for 30 min. Subsequently, the solution was filtered, and the precipitate was dried and calcined at 600°C for a duration of 2 h to yield V_2_O_5_.

2 $${\mathrm{Na}}_{{4}} {\mathrm{V}}_{{2}} {\mathrm{O}}_{{7}} + {\text{ 4NH}}_{{4}}^{ + } \to {\mathrm{2NH}}_{{4}} {\mathrm{VO}}_{{3}} + {\text{ 2NH}}_{{3}} + {\text{ 4Na}}^{ + } + {\text{ H}}_{{2}} {\mathrm{O}}$$

3 $${\mathrm{2NH}}_{{4}} {\mathrm{VO}}_{{3}} \to {\mathrm{V}}_{{2}} {\mathrm{O}}_{{5}} + {\text{ NH}}_{{3}} + {\text{ H}}_{{2}} {\mathrm{O}}$$

## Conclusions

In this study, a method for recovering vanadium from a spent vanadium catalyst (SVC) utilized in a sulfuric acid production facility in Egypt was proposed and refined. Leaching experiments were performed to extract vanadium from the spent catalyst employing various leaching agents. Parameters for leaching, including initial acid concentration, solid-to-liquid (S/L) ratio, temperature, and duration, were adjusted to enhance the leaching process. Under optimal conditions, 95% of vanadium was recovered through citric acid leaching (1 M citric acid, 2% S/L ratio, 70°C, pH 1.7, and a duration of 2 h). Vanadium leaching with citrate involves complexation, where vanadium species interact with citrate in a 2:2 ratio. To produce V_2_O_5_, forty grams of ammonium sulfate were incorporated into 300 mL of the leaching solution and stirred for 30 min; subsequently, the solution was filtered, and the resulting precipitate was dried and calcined at 600°C. These findings establish a basis for future research on the extraction of vanadium from the substantial quantities of accumulated spent catalysts and may contribute to environmental preservation through a cost-effective methodology.

## Data Availability

The datasets used and/or analyzed during the current study are available from the corresponding author on reasonable request.
